# Influence of Type 2 Diabetes on Prevalence of Key Periodontal Pathogens, Salivary Matrix Metalloproteinases, and Bone Remodeling Markers in Sudanese Adults with and without Chronic Periodontitis

**DOI:** 10.1155/2016/6296854

**Published:** 2016-02-17

**Authors:** Hasaan Gassim Mohamed, Shaza Bushra Idris, Manal Mustafa, Mutaz Faisal Ahmed, Anne Nordrehaug Åstrøm, Kamal Mustafa, Salah Osman Ibrahim

**Affiliations:** ^1^Department of Clinical Dentistry, Faculty of Medicine and Dentistry, University of Bergen, Årstadveien 19, 5009 Bergen, Norway; ^2^Department of Oral Rehabilitation, Faculty of Dentistry, University of Khartoum, Al-Qasr Street, 11123 Khartoum, Sudan; ^3^Oral Health Competence Center in Western Norway, Hordaland, Årstadveien 21, 5009 Bergen, Norway; ^4^Hamad Medical Corporation, 3050 Doha, Qatar

## Abstract

This study compared the influence of type 2 diabetes on the occurrence of six periodontal pathogens in plaque samples of patients with and without chronic periodontitis. Levels of salivary MMP-8, MMP-9, RANKL, and OPG were also investigated. The study enrolled 31 patients with type 2 diabetes and chronic periodontitis (DM + CP), 29 with chronic periodontitis (CP), and 20 with type 2 diabetes (DM). Questionnaire-guided interviews were conducted and plaque index, bleeding on probing, and pocket depth were recorded. Polymerase chain reaction (PCR) was utilized to determine the prevalence of the bacteria. The levels of salivary molecules were determined by enzyme immunosorbent assay (ELISA). The CP group had the highest prevalence of* P. gingivalis* (81.5%), followed by the DM + CP (59.3%) and DM (55.0%) groups (*P* > 0.05). Similar trends were observed for* P. intermedia* and* T. denticola*. The prevalence of* T. forsythia *was 100% in both periodontitis groups compared to 90% in the DM group. There were no significant differences between the groups regarding the concentrations of MMP-8, MMP-9, or OPG. RANKL concentrations were below the detection limit. Our data show that type 2 diabetes has no significant influence on the prevalence of the investigated periodontal pathogens, or the levels of salivary MMP-8, MMP-9, and OPG.

## 1. Introduction

Chronic periodontitis is an inflammatory condition that affects oral tissues surrounding the teeth. It is characterized by destruction of periodontal connective tissues and tooth-supporting bone accompanied by apical migration of the epithelial attachment, potentially leading to tooth loss [1]. This process is guided by the host immune-inflammatory reaction in response to putative pathogenic bacteria that are embedded in dental plaque [2]. Dental plaque is a biofilm attached to the tooth surface and harbors a complex microbiological community [3], among which some have been found to be highly associated with chronic periodontitis such as* Porphyromonas gingivalis*,* Aggregatibacter actinomycetemcomitans*,* Tannerella forsythia*,* Treponema denticola*,* Campylobacter rectus*, and* Prevotella intermedia* [4, 5].

It is now widely accepted that chronic periodontitis is one of the classical complications of diabetes [6]. There is, however, contradictory evidence about the effect of type 2 diabetes on dental plaque microbiota. Some studies have reported significant differences in the bacterial composition of dental plaque between individuals with and without type 2 diabetes [7, 8], while others failed to detect any difference [9, 10].

One of the suggested mechanisms by which hyperglycemia might influence chronic periodontitis is by interfering with the host immune-inflammatory response [11]. As part of the host response to bacterial challenge, resident and chemoattracted immune cells secrete a group of zinc-dependent endopeptidase enzymes, collectively known as matrix metalloproteinases (MMPs). These enzymes are responsible for most of the extracellular matrix degradation in both healthy and diseased tissues [12]. MMP-8 (collagenase-2) and MMP-9 (gelatinase-B) are the most common MMPs involved in periodontal tissue destruction [13]. Most of the MMPs detected in saliva are secreted by polymorphonuclear leukocytes [14]. The action of MMPs is opposed by tissue inhibitors of metalloproteinases (TIMPs); thus the imbalance between both enzymes can shape periodontal disease progression [12, 15].

A balance between osteoblasts and osteoclasts maintains the integrity of bone tissues [16]. Accordingly, bone resorption occurs if the balance is shifted towards increased osteoclast activity. Osteoclasts are activated by an osteoclast differentiation factor called receptor activator of nuclear factor-*κ*B ligand (RANKL) [17]. It is mainly secreted by activated T-cells and B-cells [18]. The action of RANKL can be blocked by osteoprotegerin (OPG), a soluble decoy receptor that competes with RANKL by binding to its receptor (RANK) on the surface of preosteoclasts and osteoclasts [19]. RANKL/OPG ratio has been reported to be a good surrogate marker for periodontitis-induced bone destruction [20]. Furthermore, it has been reported that type 2 diabetes is associated with higher gingival crevicular fluid (GCF) levels of RANKL [21]. Moreover, a positive correlation between RANKL/OPG ratios in GCF and glycated hemoglobin (HbA1c) has been also observed [22].

Saliva is a promising point-of-care diagnostic tool because it can be utilized in chair-side detection of disease markers, as it contains a vast number of locally as well as systemically expressed proteins [23]. In addition, it can be easily collected with minimum invasiveness and cost. The combined analysis of periodontitis related salivary markers and bacterial pathogens is a powerful approach for disease detection and follow-up [24].

The aim of this study was to investigate the influence of type 2 diabetes on the prevalence of six putative periodontal pathogens in subgingival plaque samples obtained from patients with and without chronic periodontitis. The impact of type 2 diabetes on levels of salivary MMP-8, MMP-9, RANKL, and OPG was also investigated. We tested the hypothesis that type 2 diabetes adversely influences the prevalence of the periodontal pathogens investigated and the levels of salivary MMP-8, MMP-9, RANKL, and OPG.

## 2. Materials and Methods

### 2.1. Study Design and Participants

In total, 80 individuals were included in this cross-sectional study representing a randomly selected subset from 461 participants included in a previous study by Mohamed et al. [25]. The subjects were stratified into three groups: 31 with type 2 diabetes and chronic periodontitis (DM + CP), 29 with chronic periodontitis (CP), and 20 with type 2 diabetes (DM). The study participants were enrolled between July and December 2012. Patients with type 2 diabetes were enrolled from the dental clinic at Jaber Abol'ez Diabetes Center in Khartoum, Sudan. Diabetes was diagnosed by specialist physicians at the center according to the criteria of the American Diabetes Association [26]. An HbA1c test was performed for patients with type 2 diabetes to determine the level of glycemic control (well-controlled, HbA1c ≤ 8%, and poorly controlled, HbA1c > 8% [21]) using a commercially available kit (LabonaCheck*™* A1c analyzer). Participants in the CP group were recruited from the outpatient dental clinic at the Khartoum Dental Teaching Hospital. Eligibility criteria for participation were (i) being diagnosed with type 2 diabetes for more than one year, for patients with diabetes [27], (ii) having at least 10 remaining teeth, (iii) no antibiotic and no steroid and/or nonsteroidal anti-inflammatory medication used during the last 3 weeks, and (iv) no immunosuppressive chemotherapy, no current acute illness, no professional periodontal treatment received during the last 6 months, and no ongoing pregnancy or lactation [28]. Questionnaire-guided interviews were conducted for all participants after enrolment [25]. Ethnicity was categorized into Afro-Arab and African tribes [29].

The study protocol was approved by the Ministry of Health in Sudan and the Norwegian Research Ethics Committee at the University of Bergen (2012/1470/REK Vest). Written informed consents were obtained from all participants and the steps of the oral clinical examination and the sampling procedures were explained. The participants were informed of their dental diagnosis and referred for appropriate dental treatment if indicated.

### 2.2. Clinical Examination

The clinical examination was performed by a single examiner (HGM). The examination included all teeth except the 3rd molars using a color-coded periodontal probe (N22, 2-4-6-8-10-12 mm markings), a color-coded Nabors furcation probe (NAB2, 3-6-9-12 mm markings), curette, mirror, probe, tweezers, and cotton rolls. Dental plaque was assessed using the Silness and Löe Index [30]. Bleeding on probing (BoP) was recorded as present or absent, and probing depths were scored as mm (from the gingival margin to the base of the periodontal pocket) at four sites per tooth (mesial, distal, buccal, and lingual). Participants were diagnosed as having chronic periodontitis if they had at least two sites with bleeding pockets of ≥4 mm (not on the same tooth) [31]. The intraexaminer reliability of the solo examiner HGM was assessed by Cohen's kappa (*κ*) to estimate coefficients of agreement of dichotomous judgments in two different sessions and *κ* was calculated for periodontal diagnosis (0.88).

### 2.3. Subgingival Plaque Samples

Four microbial samples were obtained from each participant, representing the four quadrants. The samples were collected from the 1st molar. If missing, 2nd molar, 2nd premolar, or 1st premolar was sampled, respectively. Quadrants with missing posterior teeth (premolars and molars) were excluded from the sampling. After removing the supragingival biofilm with sterile cotton pellets, the selected sites were dried and isolated with cotton rolls. A sterile paper point ISO (International Organization for Standardization) size 40 was inserted in the sulcus/pocket. Thereafter, the four paper points were pooled into one tube, labeled, and stored in liquid nitrogen for further analysis.

### 2.4. Saliva Samples

Unstimulated whole saliva samples were collected from all the study participants before the clinical examination. Participants were asked to refrain from eating or drinking for at least one hour prior to saliva collection. Donors, sitting comfortably in an upright position with their heads tilted slightly downwards, were instructed to allow saliva to collect in their mouths before gently expectorating into a sterile 10 mL tube for 5 minutes. The samples were then aliquoted and immediately stored in liquid nitrogen for further analysis.

### 2.5. DNA Purification

Bacterial DNA was extracted and purified using the MasterPure DNA purification kit according to the manufacturer's instructions (Epicentre Biotechnologies, Madison, Wisconsin). Briefly, the pooled samples were suspended in 300 *μ*L TE buffer and incubated overnight with 1 *μ*L lysozyme solution at 37°C. Next day, tissue and cell lysis solution, protease, and RNase were added. Thereafter, protein precipitation reagent was added and the mixture was centrifuged. The supernatant was collected, mixed with isopropanol, and then centrifuged. Finally, the DNA pellet was washed with ethanol and resuspended in 25 *μ*L TE buffer. The amount of DNA was measured for each sample by NanoDrop ND-1000 Spectrophotometer (NanoDrop Technologies, Wilmington, DE, USA).

### 2.6. Polymerase Chain Reaction

After DNA purification, conventional polymerase chain reaction (PCR) was performed under standard conditions to investigate the prevalence of* P. gingivalis, T. forsythia*,* P. intermedia, T. denticola, C. rectus, and A. actinomycetemcomitans*. A set of specific primers were used for the PCR [32]:* P. gingivalis*, sense, 5′-AGGCAGCTTGCCATACTGCGG-3′, and antisense, 5′-ACTGTTAGCAACTACCGATGT-3′;* T. forsythia*, sense, 5′-GCGTATGTAACCTGCCCGCA-3′, and antisense, 5′-TGCTTCAGTGTCAGTTATACCT-3′;* C. rectus*, sense, 5′-TTTCGGAGCGTAAACTCCTTTTC-3′, and antisense, 5′-TTTCTGCAAGCAGACACTCTT-3′;* P. intermedia*, sense, 5′-TTTGTTGGGGAGTAAAGCGGG-3′, and antisense, 5′-TCAACATCTCTGTATCCTGCGT-3′;* T. denticola*, sense, 5′-TAATACCGAATGTGCTCATTTACAT-3′, and antisense, 5′-TCAAAGAAGCATTCCCTCTTCTTCTTA-3′; and* A. actinomycetemcomitans*, sense, 5′-AAACCCATC-TCTGAGTTCTTCTTC-3′, and antisense, 5′-ATGCCAACTTGACGTTAAAT-3′.

**Figure 1:**
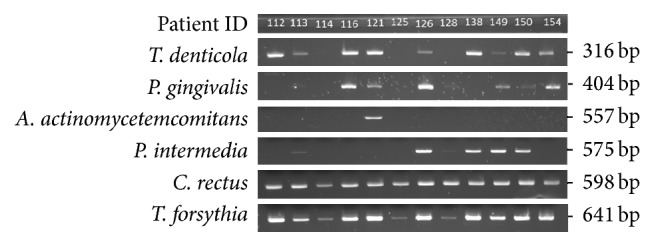
Representative results of electrophoresis of PCR products from dental plaque samples, patients ID 112 to 154.

The final reaction volume (50 *μ*L) consisted of 100 *μ*M dNTP, 0.4 *μ*M of each primer, 1.75 mM MgCl_2_, 1 U of AmpliTaq Gold® DNA Polymerase (Applied Biosystems, Foster City, CA, USA), and 50 ng of extracted DNA. The target genes were amplified in a thermocycler (GeneAmp PCR System 9700, Applied Biosystems, Foster City, CA, USA) as follows: one cycle at 95°C for 10 minutes, 40 cycles at 95°C for 30 seconds for denaturation, 62°C for 30 seconds for annealing, 72°C for 1 minute for extension, and a final extension of 72°C for 10 minutes. The annealing temperature was adjusted for* A. actinomycetemcomitans* to 60°C. Negative control (master mix without DNA template) was included in each PCR run. The PCR products were loaded and separated by agarose gel electrophoresis (2.2% FlashGel DNA System; Lonza, Walkersville, MD). The stained DNA bands were visualized by ultraviolet light and the data were reported as present or absent (Figure 1).

### 2.7. Enzyme Linked Immunosorbent Assay

Frozen saliva samples were thawed and centrifuged at 14000 rpm for 15 minutes and the supernatants were collected. The levels of MMP-8, MMP-9, OPG (Sigma-Aldrich, St. Louis, MO, USA), and RANKL (MyBioSource, CA, USA, and PeproTech EC, London, UK) were determined using enzyme linked immunosorbent assays (ELISA) according to the manufacturers' instructions. Optical densities were determined using a microplate reader (FLUOstar OPTIMA, BMG Labtech, Germany). The final concentrations are presented in pg/mL.

### 2.8. Statistical Analysis

Potential differences in demographic and clinical indicators between the study groups were tested using chi-square and Fisher's exact test for categorical variables, independent sample *t*-test and one-way ANOVA for normally distributed variables, and Mann-Whitney *U* test and Kruskal-Wallis test for skewed data. Frequencies of detection of the microbes under investigation are expressed in (%), and chi-square test or Fisher's exact test was used to examine the statistical differences between the study groups. The concentrations of the detected salivary molecules were compared by Kruskal-Wallis test. Adjustment for the potentially confounding effect of age, gender, smoking status, and ethnicity was done by logistic regression analysis for the prevalence of bacteria and by generalized linear models (GLM) with Gaussian family and log function for concentrations of salivary markers. Stata 13 (Stata Corp. 2013, Stata Statistical Software: Release 13; College Station, TX: StataCorp LP) was used for statistical analysis. *P* values less than 0.05 were considered statistically significant and were adjusted for multiple comparisons when indicated.

## 3. Results

The distributions of the demographic and clinical indicators across the study groups are presented in Table 1. The age of the study participants ranged from 24 to 70 years. Comparisons of the clinical periodontal parameters between the study groups revealed that the DM + CP group had higher dental plaque index and more pocket depth (≥6 mm) than the CP group (*P* < 0.05). Moreover, the DM + CP group had more BoP than both the CP and DM groups (*P* < 0.01).

The CP group had the highest prevalence of* P. gingivalis* (81.5%), followed by DM + CP (59.3%) and DM (55.0%) groups (*P* > 0.05). The same trend was observed for* P. intermedia* and* T. denticola*. The prevalence of* T. forsythia* was 100% in both periodontitis groups (DM + CP and CP) compared to 90% in the DM group.* C. rectus* was detected in plaque samples of all the study participants. All plaque samples in the DM group were negative for* A. actinomycetemcomitans*, while the prevalence in the DM + CP and CP groups was 7.4% and 11.1%, respectively (Table 2).

**Figure 2:**
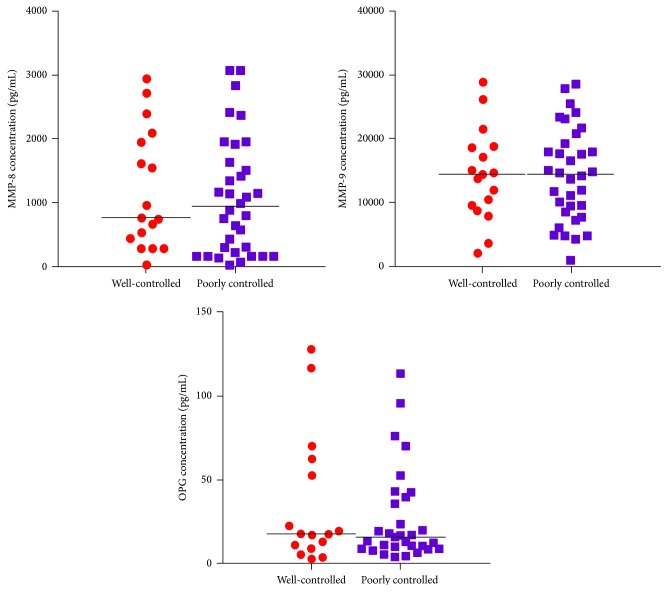
Distribution of salivary MMP-8, MMP-9, and OPG concentrations (pg/mL) in well-controlled (*n* = 37) and poorly controlled (*n* = 17) type 2 diabetes patients.

Comparisons of salivary MMP-8, MMP-9, and OPG across the study groups were not statistically significant (Table 3). Nonetheless, there was a trend towards increased concentration of MMP-8 in the chronic periodontitis groups (DM + CP and CP) compared to the DM group. In addition, the levels of the molecules under investigation did not differ significantly between well-controlled (*n* = 34) and poorly controlled (*n* = 17) type 2 diabetes patients (Figure 2). The concentrations of RANKL were below the detection limit in all saliva samples.

**Figure 3:**
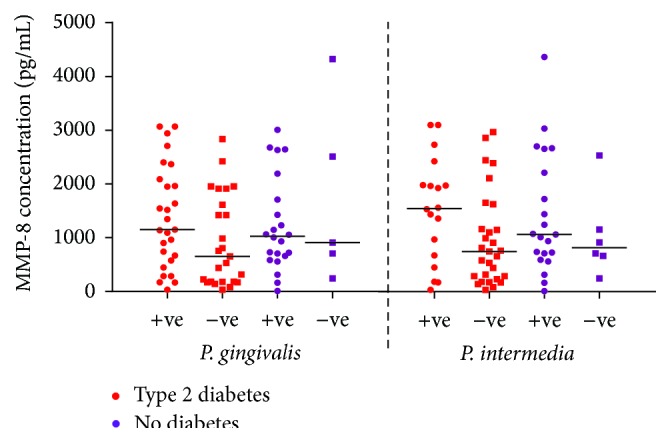
Distribution of salivary MMP-8 concentrations (pg/mL) according to the prevalence of* P. gingivalis* and* P. intermedia* in subjects with and without type 2 diabetes regardless of the periodontal status (*n* = 80).

**Figure 4:**
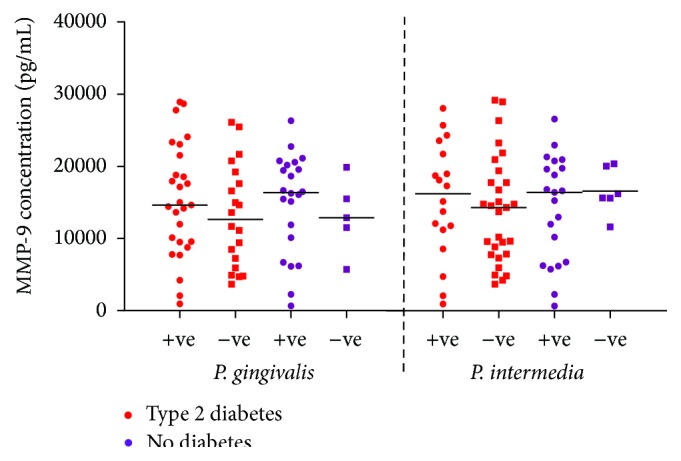
Distribution of salivary MMP-9 concentrations (pg/mL) according to the prevalence of* P. gingivalis* and* P. intermedia* in subjects with and without type 2 diabetes regardless of the periodontal status (*n* = 80).

**Figure 5:**
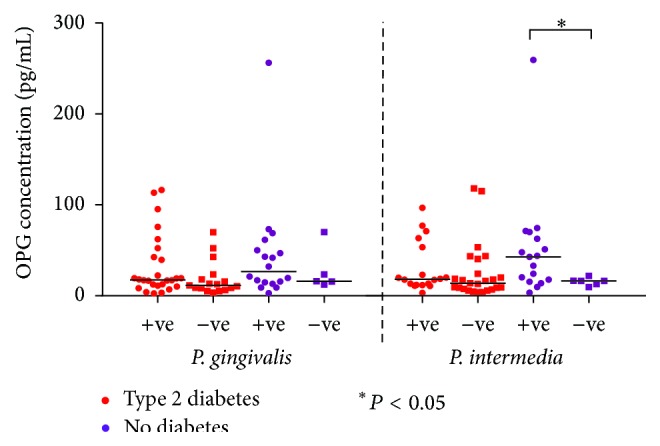
Distribution of salivary OPG concentrations (pg/mL) according to the prevalence of* P. gingivalis* and* P. intermedia* in subjects with and without type 2 diabetes regardless of the periodontal status (*n* = 80).

Distributions of salivary MMP-8, MMP-9, and OPG concentrations according to the prevalence of* P. gingivalis* and* P. intermedia* are presented in Figures 3–5 among subjects with and without type 2 diabetes regardless of the periodontal status (*n* = 80). Regardless of the diabetic status, MMP-8 levels remained higher in subjects with positive* P. gingivalis* and* P. intermedia* scores than in those with negative scores, albeit not statistically significant (Figure 3). The levels of MMP-9 followed the same pattern as MMP-8 for* P. gingivalis* results. On the other hand, subjects without diabetes had similar MMP-9 level comparing those with positive and negative* P. intermedia* scores (Figure 4). In patients with type 2 diabetes, the concentration of OPG did not discriminate between those with and without* P. gingivalis* or* P. intermedia* in their plaque samples, while for those without diabetes, individuals with positive* P. gingivalis* and* P. intermedia* scores tend to have higher concentrations of OPG (Figure 5).

## 4. Discussion

Periodontal pathogens are considered as a triggering factor of the disease [33]. Recently, it was suggested that periodontal tissue destruction is mediated by the host inflammatory response when the balance in the relative quantities of the existing bacteria in dental plaque is disturbed [34, 35]. The pathogens that are responsible for this imbalance are called “keystone pathogens” [36].

In the present study, the DM + CP group had worse clinical periodontal parameters such as plaque index and BoP compared to the other groups. These findings are in accordance with those of several earlier studies reporting compromised periodontal parameters among patients with type 2 diabetes [37, 38].

Most of the investigated microbes were more prevalent in individuals with chronic periodontitis (DM + CP and CP) compared to the DM group. In addition, the effect of type 2 diabetes on the prevalence of the investigated bacteria was not significant. Similar findings were reported by others using different methodological approaches [10, 39]. Field et al. [10] reported that* P. gingivalis* is significantly higher in chronic periodontitis patients with and without type 2 diabetes than in type 2 diabetes patients without periodontitis, which is in line with our findings, although the difference in our patients was not statistically significant. Moreover, our results demonstrated that 30% of the plaque samples obtained from participants with chronic periodontitis scored negative for* P. gingivalis* (data not shown). Although* P. gingivalis* is one of the keystone pathogens in chronic periodontitis, it is not always detectable in periodontally diseased sites [40].

In the present study, the prevalence of* A. actinomycetemcomitans* was relatively low in all the study groups. Elabdeen et al. [41] investigated subgingival microorganisms in Sudanese patients (with an age range of 13–30 years) with aggressive periodontitis using DNA-DNA hybridization (checkerboard) technique. They reported relatively low levels of* A. actinomycetemcomitans* in aggressive periodontitis patients as well as in healthy controls. Additionally, studies among patients with chronic periodontitis from Italy [42], Thailand [43], and Japan [44] reported low prevalence of* A. actinomycetemcomitans*. In that regard, a study from China demonstrated a low prevalence of* P. intermedia* in patients with type 2 diabetes and chronic periodontitis when compared to systemically healthy individuals with chronic periodontitis [45]. The same trend was observed in the present study. It is noteworthy that periodontal microbiota may vary according to the geographical areas, which makes comparisons in that regard between studies from different geographical backgrounds rather difficult [4].

The role of MMP-8 and MMP-9 in periodontal tissue destruction is well established [12]. In the present study, as in a study from another group [46], there were no significant differences in the levels of salivary MMP-8 and MMP-9 between the study groups. Another study reported no significant difference comparing MMP-8 in GCF between individuals with and without type 2 diabetes [47], while others reported higher MMP-8 in saliva of patients with type 2 diabetes than in subject without the disease [48, 49]. Moreover, Javed et al. [50] investigated the level of salivary MMP-8 among prediabetic patients. They concluded that MMP-8 levels did not differ significantly between chronic periodontitis subjects with and without prediabetes.

In the present study, there was a trend towards increased OPG levels in both the DM + CP and CP groups compared to the DM group. This can be attributed to the fact that OPG levels increase in response to increased osteoclastogenesis [51]. Moreover, our data demonstrate that the level of glycemic control did not affect the concentrations of molecules under investigation. A follow-up study investigating the GCF levels of RANKL and OPG over 6 months found no difference in OPG concentrations between well-controlled and poorly controlled type 2 diabetes patients at any time point, while RANKL remained significantly higher in the poorly controlled group at all the study time points [22].

The association between the systemic levels of OPG and the nonoral medical complications of type 2 diabetes is well documented [52, 53]. In contrast, information about its levels in oral fluids of type 2 diabetes patients and its association with periodontal disease is scarce. Costa et al. [48] reported elevated levels of salivary OPG in patients with type 2 diabetes compared to systemically healthy subjects with chronic periodontitis. Another study among patients with type 1 diabetes concluded that those with diabetes had higher plasma concentration of OPG and lower RANKL/OPG ratio compared to individuals without diabetes [54]. In contrast, an experimental study among rats demonstrated that type 1 diabetes influences periodontal bone tissues by decreasing OPG and increasing the RANKL/OPG ratio [55]. In this regard, the RANKL/OPG ratio is a good indicator of bone tissue destruction induced by periodontitis [20]. Nevertheless, it was not possible to calculate the RANKL/OPG ratio in the present study because the concentrations of RANKL were below the detection limit, although two commercially available kits were used for the RANKL detection.

The study design of the present study (cross-sectional) might contribute to the absence of association observed between type 2 diabetes and the investigated salivary molecules and periodontal pathogens, as it captures a single time point of the ongoing inflammatory process. In the present study, periodontal pocket depth and bleeding on probing were used to define cases with chronic periodontitis. Both periodontal parameters reflect current disease status and have been reported to be associated with local inflammatory activity as well as ecological changes at sample sites [56, 57]. The exclusion criterion for the use of antibiotics (during the last 3 weeks) might be considered as a short period for the effect of antibiotics to be diminished which might in turn influence our results.

## 5. Conclusions

Within the limitations of the present study, our data suggest that type 2 diabetes has no significant influence on the prevalence of the investigated periodontal pathogens. Moreover, we were unable to detect a significant difference in levels of salivary MMP-8, MMP-9, and OPG between individuals with and without type 2 diabetes. Large follow-up studies are needed to trace the potential effect of type 2 diabetes on bacterial composition of dental plaque and on bone remodeling markers in oral fluids.

## Figures and Tables

**Table 1:** Distribution of sociodemographic and clinical indicators.

Variable	DM + CP (*n* = 31)	CP (*n* = 29)	DM (*n* = 20)
Age, mean (SE)^1^	53.55 (1.82)	52.03 (1.34)	51.15 (2.39)
Gender, % (*n*)^2^			
Male	48.40 (15)	48.30 (14)	30.00 (6)
Female	51.60 (16)	51.70 (15)	70.00 (14)
Ethnicity, % (*n*)^3^			
Afro-Arab	93.55 (29)	82.76 (24)	63.16 (12)
African	6.45 (2)	17.24 (5)	36.84 (7)
Education, % (*n*)^2^			
Illiterate	25.80 (8)	34.50 (10)	25.00 (5)
Literate	74.20 (23)	65.50 (19)	75.00 (15)
Employment, % (*n*)^2^			
Unemployed	51.60 (16)	51.70 (15)	75.00 (15)
Employed	48.40 (15)	48.30 (14)	25.00 (5)
Smoking, % (*n*)^3^			
Yes	16.10 (5)	31.00 (9)	10.00 (2)
No	83.90 (26)	69.00 (20)	90.00 (18)
Hypertension, % (*n*)^2^			
Yes	29.00 (9)	17.20 (5)	30.00 (6)
No	71.00 (22)	82.80 (24)	70.00 (14)
Regular dental attendance, % (*n*)^3^			
Yes	0.00 (0)	6.90 (2)	10.00 (2)
No	100.00 (31)	93.10 (27)	90.00 (18)
Duration of diabetes-years, mean (SE)^4^	8.40 (1.09)	—	10.50 (1.99)
HbA1c%, mean (SE)^5^	9.17 (0.34)	—	9.37 (0.52)
Plaque index, mean (SE)^1^	1.68 (0.07)^a^	1.42 (0.06)^b*∗*^	1.47 (0.06)^ab^
Percentage of teeth with BoP, mean (SE)^6^	56.95 (3.71)^a^	22.21 (2.50)^b*∗∗*^	29.50 (3.99)^b*∗∗*^
Pocket depth, mean (SE)^4^	4.18 (0.05)	4.25 (0.09)	—
Pocket depth, % (*n*)^3^			
4-5 mm	58.10 (18)	86.20 (25)^*∗*^	—
≥6 mm	41.90 (13)	13.80 (4)	—

Different letters indicate statistically significant differences.

^1^One-way ANOVA.

^2^Chi-square test.

^3^Fisher's exact test.

^4^Mann-Whitney *U* test.

^5^Independent sample *t*-test.

^6^Kruskal-Wallis test.

^*∗*^ *P* < 0.05.

^*∗∗*^ *P* < 0.01.

**Table 2:** Prevalence of periodontal pathogens detected by PCR (*n* = 74).

Bacteria, % (*n*)	DM + CP (*n* = 27)	CP (*n* = 27)	DM (*n* = 20)
*P. gingivalis* ^1^	59.3 (16)	81.5 (22)	55.0 (11)
*P. intermedia* ^1^	44.4 (12)^b*∗*^	77.8 (21)^a^	30.0 (6)^b*∗∗*^
*T. forsythia* ^2^	100.0 (27)	100.0 (26)^†^	90.0 (18)
*T. denticola* ^2^	88.9 (24)	100.0 (27)	70.0 (14)
*C. rectus* ^2^	100.0 (27)	100.0 (27)	100.0 (20)
*A. actinomycetemcomitans* ^2^	7.4 (2)	11.1 (3)	0.0 (0)

Different letters indicate statistically significant differences, adjusting for age, gender, smoking status, and ethnicity.

^1^Chi-square test.

^2^Fisher's exact test.

^†^One sample was excluded from the analysis.

^*∗*^ *P* < 0.05.

^*∗∗*^ *P* < 0.01.

**Table 3:** Concentrations (pg/mL) of the detected inflammatory molecules by ELISA (*n* = 80).

Study group	Concentration	GLM^*∗∗*^			
	Mean (SE)^*∗*^	Coefficient	SE	95% CI	*P* value
MMP-8					
DM + CP	1256.27 (166.65)	0.22	0.28	(−0.32, 0.77)	0.42
CP	1305.30 (193.85)	0.34	0.27	(−0.19, 0.87)	0.21
DM	923.35 (194.74)	Reference group			
MMP-9					
DM + CP	16188.39 (1303.30)	0.24	0.16	(−0.08, 0.56)	0.14
CP	14147.76 (1274.33)	0.15	0.17	(−0.17, 0.48)	0.36
DM	11378.75 (1401.43)	Reference group			
OPG					
DM + CP	33.51 (7.11)	0.43	0.57	(−0.69, 1.55)	0.45
CP	39.91 (10.02)	0.72	0.55	(−0.36, 1.79)	0.12
DM	23.53 (4.26)	Reference group			

^*∗*^Kruskal-Wallis test.

^*∗∗*^Generalized linear models with Gaussian family and log function adjusting for age, gender, smoking status, and ethnicity.
